# Sharing science: a new era of widening access to research in reproductive medicine

**DOI:** 10.1093/hropen/hox004

**Published:** 2017-03-29

**Authors:** Siladitya Bhattacharya

Welcome to Human Reproduction Open. As the first fully open access ESHRE journal to join a group of highly successful print and online allied journals, HROpen seeks to deliver a journal for the future, with the intention of informing and engaging a new type of reader.

The 21st century communication is all about access and immediacy. The traditional paper-based scientific journal is limited by the technology which created it over 600 years ago—the printing press. The internet has raised expectations in all aspects of life. Science and medicine are no different. No longer are people prepared to wait for knowledge to be collated and released in the neat packages of journal issues. HROpen will ensure that they do not have to.

Scholarship is not—and should not—be the preserve of the exclusive few who can afford subscription charges. Among those who have been too often shut out by the existing models are scholars without the support of wealthy institutions (often in developing countries) and lay audiences (often patients and their relatives). Open access journals in general, and HROpen in particular, hope to bring research and evidence to groups like these, who often have so much to benefit from it.

Do we really need another journal in Reproductive Medicine? We will always need tools which are more efficient, agile, environment friendly and customized to our needs. We are lucky to be living at a time of accelerated scientific discovery and output covering the full spectrum of research in our field, from laboratory science through translational medicine to social science, ethics and philosophy. In HROpen, we offer a high-quality open access journal in reproductive medicine which harnesses the full potential of the internet to deliver scientific knowledge which is current, personalized and fit for purpose. Our vision is to create a shared repository for scientific and clinical knowledge for patients and clinicians which can facilitate joint decision-making.

As in journalism, academic publishing tends to favour novelty and is biased towards informing its readers what is new. Yet, with relatively few exceptions, knowledge advances slowly, in incremental stages, through debate, challenge and ultimately acceptance. Scientists have always been aware of this. When, in 1676, Isaac Newton remarked ‘If I have seen further, it is by standing on the shoulders of giants’, what he meant was that getting to the truth was an iterative process, building on previous advances. While discovery is critical to all scientific thought, we are beginning to accept the fact that validation of the truth through replication is just as important. As curators of scientific knowledge, journals owe it to their readers to not only report what is new but also whether these new findings are valid in other settings and laboratories and fit for clinical application. Replication is hard and can be frustrating, not least because the results can be difficult to publish, but it remains an important component of scientific advance. Sign posting in science is becoming increasing popular—but while most randomized trials are registered and their protocols published, this is not the norm for laboratory-based studies. HROpen will publish high-quality research which is scientifically sound, advances the literature on a clinically relevant topic and is worthy of publication even where it may not be the first of its kind. The journal will also be open to submissions that enhance scientific thought as well as governance by publishing research protocols and pilot studies alongside phase three trials and high-quality systematic reviews. To put these findings in wider perspective, HROpen will educate, stimulate and challenge its readers through a series of commissioned reviews by respected experts in the field.

The purpose of reproductive medicine research must be to improve clinical care through greater understanding of the underlying biological processes and designing interventions which are safe, and effective. Our patients are increasingly conducting their own research into their conditions and attempting to identify strategies which are effective and consistent with their own values and preferences. Their searches are not fundamentally different from those undertaken by clinicians and readers of this journal but they remain fundamentally disconnected. Navigating headlines and search engine results without the skills needed to separate opinion from scientific fact can often lead to incomprehension, frustration and lack of trust in the medical care provider. At a time when all major funding bodies are insistent on patient involvement in the planning and execution of research, it is fundamental that research output should be available to patients in a format which is simple, unbiased and context dependent. HROpen is the first journal in our field to offer a lay summary of new research papers explaining the meaning and relevance of the science. We hope that the open access format will allow patients to access these summaries and empower them to decide for themselves whether each paper highlights an association, offers convincing proof of causality or the success of an intervention.

HROpen will publish well-conducted studies addressing clinical, biological, environmental, ethical and social aspects of reproductive medicine in its wider sense—beyond the relatively narrow confines of infertility and assisted reproduction. Our primary aim is to increase global access to scientific thought in our field and break down the barriers between formal scientific publication and sources of clinical information for lay people. We wish to venture beyond the narrow confines of traditional metrics and are keen to embrace a variety of alternative ways of assessing the reach and impact of our articles. We strongly believe that to have genuine impact, scholarship must be made available to all, in a format which is accessible for all. I believe that HROpen will be able to do just that.

